# Zonal image analysis of tumour vascular perfusion, hypoxia, and necrosis

**DOI:** 10.1038/sj.bjc.6600343

**Published:** 2002-06-05

**Authors:** B M Fenton, S F Paoni, B K Beauchamp, I Ding

**Affiliations:** Department of Radiation Oncology, University of Rochester Medical Center, Rochester, New York, NY 14642, USA

**Keywords:** hypoxia markers, image processing, immunohistochemistry, tumour oxygenation

## Abstract

A number of laboratories are utilising both hypoxia and perfusion markers to spatially quantify tumour oxygenation and vascular distributions, and scientists are increasingly turning to automated image analysis methods to quantify such interrelationships. In these studies, the presence of regions of necrosis in the immunohistochemical sections remains a potentially significant source of error. In the present work, frozen MCa-4 mammary tumour sections were used to obtain a series of corresponding image montages. Total vessels were identified using CD31 staining, perfused vessels by DiOC_7_ staining, hypoxia by EF5/Cy3 uptake, and necrosis by haematoxylin and eosin staining. Our goal was to utilise image analysis techniques to spatially quantitate hypoxic marker binding as a function of distance from the nearest blood vessel. Several refinements to previous imaging methods are described: (1) hypoxia marker images are quantified in terms of their intensity levels, thus providing an analysis of the gradients in hypoxia with increasing distances from blood vessels, (2) zonal imaging masks are derived, which permit spatial sampling of images at precisely defined distances from blood vessels, as well as the omission of necrotic artifacts, (3) thresholding techniques are applied to omit holes in the tissue sections, and (4) distance mapping is utilised to define vascular spacing.

*British Journal of Cancer* (2002) **86**, 1831–1836. doi:10.1038/sj.bjc.6600343 www.bjcancer.com

© 2002 Cancer Research UK

## 

The relationship between tumour hypoxia and therapeutic response has been well documented in the literature for quite some time, and direct measures of tumour oxygenation have been shown to correlate with both long-term survival (Hockel *et al*, 1996) and the occurrence of distant metastases (Brizel *et al*, 1996). Two of the more prominent techniques for measuring tumour hypoxia are: (1) the Eppendorf electrode for determination of tumour pO_2_ levels, and (2) nitroimidazole hypoxia markers, e.g., EF5, NITP, and pimonidazole, which covalently bind to hypoxic tumour cells and allow immunohistochemical or flow cytometric determination of hypoxia distributions (Koch *et al*, 1995; Hodgkiss and Wardman, 1992; Raleigh *et al*, 1987). The presence of tumour necrosis can have substantial effects on each of these types of measurements. With the Eppendorf technique, necrotic regions result in a reduction in measured pO_2_ levels that is not reflective of a corresponding reduction in clonogenic survival of the tumour cells (Fenton *et al*, 1995). This leads to overestimates of the fraction of radiobiologically resistant tumour cells. In the case of hypoxia markers, regions of necrosis can also lead to inaccurate predictions in overall tumour hypoxia. Since these drugs are not metabolized in necrotic areas, such regions appear well oxygenated and lead instead to an underestimate of overall tumour hypoxia.

Several recent studies have described methods for quantifying hypoxia marker distribution as a function of distance from either perfused or anatomical blood vessels. The first (Rijken *et al*, 2000) is an elegant, multiparameter analysis of vascularity, perfusion, and hypoxia that characterises uptake of two hypoxia markers, NITP and pimonidazole, in relation to perfused vasculature. The second (Vukovic *et al*, 2001) focuses primarily on the spatial distribution of EF5 in relation to total vasculature. A potential limitation in each of these studies is the characterisation of hypoxia marker labelled cells as either positive or negative, thus ignoring any information regarding relative intensity of the marker. Since hypoxia clearly increases continuously with increasing distance from blood vessels, such a conversion of hypoxia marker intensity levels to binary images results in the loss of potentially important information regarding intermediate regions of hypoxia.

The current work presents several refinements of earlier methods: (1) Hypoxia marker images are quantified in terms of their intensity levels, thus providing an analysis of the rate at which hypoxia increases with increasing distances from blood vessels. This measurement is directly related to oxygen consumption by the tumour tissue and provides an estimate of intravascular oxygen levels. (2) Improved image analysis techniques are described for defining zonal imaging masks, thereby permitting spatial sampling of immunohistochemical images at precisely defined distances from blood vessels. (3) Thresholding techniques are described for removing artifactual holes in the tissue sections. (4) Distance mapping is used to define vascular spacing. Although previous studies (Vukovic *et al*, 2001; Rijken *et al*, 2000) have used adjacent sections for the removal of gross necrosis, the current study utilized haematoxylin and eosin staining of the same frozen sections used for hypoxia marker and vascular imaging.

## MATERIALS AND METHODS

### Tumour model

Cells from MCa-4 murine mammary carcinomas were inoculated into the mammary fat pads of C3H/HeJ mice. Guidelines for the humane treatment of animals were followed as approved by the University Committee on Animal Resources and meet the standards required by the UKCCCR guidelines (Workman *et al*, 1998).

### DiOC_7_ perfusion marker and EF5 hypoxic marker

To visualise blood vessels open to flow, an intravascular injected stain, DiOC_7_, was injected 1 min prior to tumour freezing (Trotter *et al*, 1989). This agent has been shown to provide optimal visualisation of tumour blood vessels by preferentially staining cells immediately adjacent to the vessels (Fenton *et al*, 1999). Localised areas of tumour hypoxia were assessed in 9 μm frozen tissue sections by immunohistochemical identification of sites of 2-nitroimidazole metabolism as described previously (Fenton *et al*, 1999). A pentafluorinated derivative (EF5) of etanidazole was injected i.v. 1 h before tumour freezing, at which time the EF5 has been shown to be well distributed throughout even poorly perfused regions of the tumour (Fenton *et al*, 2001a). Regions of high EF5 metabolism were visualised immunohistochemically using a fluorochrome (Cy3, Amersham) conjugated to the ELK3-51 monoclonal antibody. This antibody is extremely specific for the EF5 drug adducts that form when the drug is incorporated by hypoxic cells (Lord *et al*, 1993). Both the EF5 (made by the NCI) and the ELK3-51 were obtained from the University of Pennsylvania Imaging Service Center (C Koch, Director).

### Immunohistochemistry and image acquisition

Tumour sections were imaged using a Nikon microscope (20× objective), digitised (FlashPoint frame grabber and Sony DXC9000 3CCD camera), background-corrected, and image-analysed using Image-Pro software (version 4.5, Media Cybernetics, Silver Spring, MD, USA) with a 800 MHz Pentium computer, as previously described (Fenton *et al*, 1999). Colour images from the same 16 adjacent microscope fields were automatically acquired and digitally combined under four different staining conditions, using a Prior computer interfaced stage and Prior controller to revisit the same stage co-ordinates. First, epi-illumination images of the fluorescent green DiOC_7_(3) staining were obtained immediately after the sections were sliced on the cryostat. Following immunohistochemical staining, the tumour section was returned to the same stage co-ordinates, and fluorescent red–orange images were acquired of the distribution of the EF5/Cy3. Next, using transmitted light, matching brownish–red montages of the CD31 endothelial staining were acquired. Finally, sections were stained for haematoxylin and eosin and again imaged at the same coordinates.

### Image processing techniques

**Figure 1:**
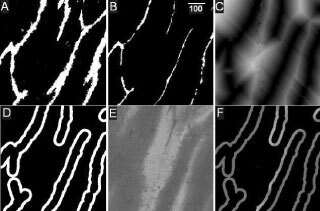
Zonal image analysis procedures: (**A**) binary image of total blood vessels (CD31 stained vessels are shown in white), (**B**) binary image of perfused vessels (DiOC_7_ vessels shown in white, scale=100 μm), (**C**) distance map of perfused blood vessels, (**D**) zonal mask of pixels 21–40 μm from perfused vessels, (**E**) EF5/Cy3 staining (lighter shades correspond to increased tumour hypoxia), (**F**) logical ‘AND’ of the zonal mask and EF5/Cy3 images, which selectively samples the distribution of EF5/Cy3 intensities within this specific zone.

To quantitate microregional EF5/Cy3 intensities variations as a function of distance from perfused blood vessels, methods somewhat similar to those of Rijken *et al* (2000) were utilised. As summarised in Figure 1

, CD31 (total blood vessels) and DiOC_7_ (perfused blood vessels) stained images were enhanced by first using colour segmentation to identify blood vessels (Fenton *et al*, 1999). For CD31 staining, specific intensity thresholds (using the HSI colour model) were interactively selected and accumulated to obtain optimal discrimination of vessels and stroma, and a binary image of the selected colours was created (Figure 1A). For DiOC_7_ staining, the Image-Pro ‘automatic bright’ thresholding was used (Figure 1B). For counting either perfused or total vessels, objects of area less than 10 μm^2^ (roughly 3.5 μm^2^ in diameter, to eliminate nonvascular artifacts) were removed, and an area of interest was outlined to omit sectioning artifacts and normal tissue. This binary image was next inverted and a ‘distance filter’ applied, which replaces the intensity of each pixel with an intensity proportional to the distance of that pixel from the nearest vessel (Figure 1C). Thus, pixels immediately adjacent to the vessels were assigned intensity 1 in the new image, and the intensities of more distant pixels increase by one grey level for each one pixel increase in distance from the vessel edge. This distance filtered image was then successively thresholded and binarized to select regions of the image within specific distances from vessels. For example, thresholding between 1–20 grey levels selects a zone 1–20 pixels away from a vessel, 21–40 selects a zone from 21–40 pixels away from a vessel, and so on. Figure 1D illustrates the binary image resulting from thresholding between grey levels of 21–40, which selects a region within approximately 21–40 microns of the nearest vessel, since each pixel is approximately one micron square.

For the EF5/Cy3 images (Figure 1E), holes in the immunohistochemical sections were automatically removed by interactively thresholding on the image to convert the pixel intensities of holes in the tissue section to zero. Finally, the binary ‘mask’ (Figure 1D) was combined with the corresponding EF5/Cy3 image (Figure 1E), using the logical ‘AND’ to obtain an image in which only those regions of the EF5/Cy3 image within the 21–40 μm zone are included (Figure 1F). This allows the determination of the EF5/Cy3 intensity distribution within a precisely defined distance from the blood vessels. Using the same sequence of steps, EF5/Cy3 intensities within each successive concentric zone were also obtained and median intensity levels were plotted as a function of distance from the nearest perfused or total vessel.

Regions of gross necrosis were selected visually from the haematoxylin and eosin images and outlined using the multiple area of interest (AOI) tool. Regions inside or outside of the AOI were converted to a binary mask (set to black or white, respectively), and percent gross necrosis was determined by the Image-Pro intensity ‘range statistics’ function. Finally, necrosis correction was applied to the zonal analysis by performing a logical ‘AND’ on the binary necrosis image and the corresponding zonal EF5/Cy3 image (Figure 1F). This converts necrotic intensities to zero while leaving other intensities unaltered, and the zero intensity regions are excluded from the analysis.

## RESULTS

Figure 2

**Figure 2:**
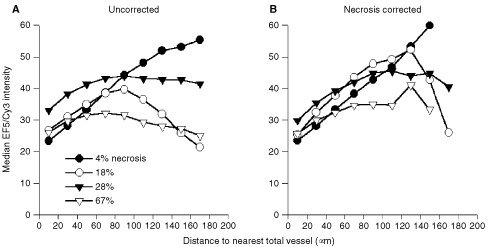
Effect of necrosis on median EF5/Cy3 intensities as a function of distance from nearest total blood vessel (CD31 staining). Each curve is based on the combination of four image montages from a single untreated tumour, and percentages of gross necrosis and tumour volumes are as follows: filled circles=4%, 520 mm^3^; open circles=18%, 900 mm^3^; filled triangles=28%, 1150 mm^3^; open triangles=67%, 1770 mm^3^. Intensities are presented as the median intensity within each 20 μm wide zone.

presents EF5/Cy3 intensities as a function of distance from the nearest total blood vessel, each point representing the median EF5/Cy3 intensity within a specific 20 μm wide zone (as detailed in Materials and Methods). The four curves in each panel correspond to MCa-4 tumours of increasing tumour volume, with correspondingly increasing percentages of gross necrosis. The slopes of these curves are directly related to the oxygen consumption of the surrounding tumour cells. Thus, steeper slopes, which are indicative of a rapid increase in hypoxia with increasing distance from the perfused vessels, correspond to increased consumption rates. Median EF5/Cy3 intensities of the innermost zone (1–20 μm from the vessels) are most reflective of the adjacent intravascular oxygen levels.

In Figure 2A, the filled circles correspond to a tumour of 4% gross necrosis, and median EF5/Cy3 intensities increase progressively with increasing distance from the nearest total blood vessel (reflecting the expected increase in tumour hypoxia with increasing distance from a vessel). With increasing percentages of necrosis, the curves of Figure 2A become less predictable, and in some cases tend to become less rather than more hypoxic at the longest distances from the vessels. In Figure 2B, gross necrotic regions have been removed as detailed in the Materials and Methods, and the analyses of Figure 2A have been repeated. Although the curves are altered somewhat by this correction, the unexpected decrease in hypoxia at the highest distances was still observed for some tumours (in particular the open circles). This decrease is possibly due to the influence of regions of necrosis that were too small to be included in the gross necrosis correction, which would be more likely to be found near nonfunctional vessels than near perfused blood vessels. In addition, the fraction of the total tumour area included in a given zone decreases at increasing distances from the vessels, due to the overlap of adjacent zones from nearby vessels (especially for the total vessels, which are more densely distributed). At distances greater than 140 μm, zones around total vessels can encompass less than 1% of the total tumour area, leading to an increase in sampling variability in these zones.

**Figure 3:**
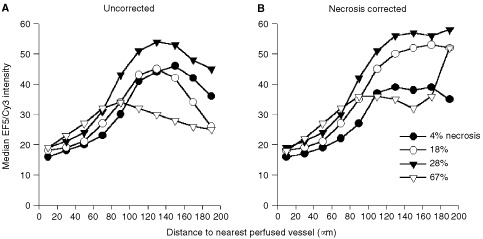
Effect of necrosis on median EF5/Cy3 intensities as a function of distance from nearest perfused blood vessel (DiOC_7_ staining). Each curve is based on images from the same untreated tumours of Figure 2. See Figure 2 for legend.

Figures 3A and B depict analyses similar to those of Figure 2, but plotted as a function of distance from perfused vessels rather than total blood vessels. Compared with Figure 2A, the median intensities for the 0–20 zone (nearest the blood vessels) in Figure 3A

are decreased. This shift is due to the inclusion of nonfunctional vessels in the total vessel plots of Figure 2A. Tumour cells around these vessels will more likely be hypoxic, and the shift between the total and perfused intensity plots is therefore a reflection of vessel functionality in a given tumour. Thus the larger intensity shift for the tumour represented by the solid triangles is suggestive of a higher proportion of nonfunctional vessels in this particular tumour, compared with the remaining three tumours. For the uncorrected analyses (Figure 3A), all tumours showed a distinct peak in EF5/Cy3 intensities at distances between 100–150 μm, followed by a distinct decline. After necrosis correction (Figure 3B), the shape of each of these curves was dramatically altered such that EF5/Cy3 intensities generally tended to plateau at distances of about 120–130 μm from the nearest vessel.

Figure 4

**Figure 4:**
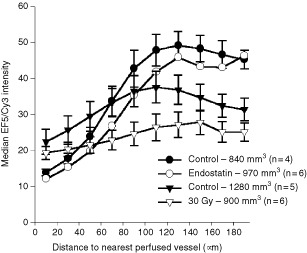
Effect of tumour volume and treatment on median EF5/Cy3 intensities as a function of nearest perfused (DiOC_7_ staining) blood vessel (mean±s.e.m., based on 4–6 tumours per group). filled circles=840 mm^3^ controls, open circles=970 mm^3^ endostatin treated tumours, filled triangles=1280 mm^3^ controls, open triangles=900 mm^3^ 30 Gy irradiated tumours (none corrected for necrosis). For clarity, the s.e.m. are omitted for the 30 Gy and 1280 mm^3^ control tumours.

presents the effects of tumour growth, antiangiogenic treatment, and irradiation, to illustrate the influence of different pathophysiological alterations on zonal variations in hypoxia. Untreated controls are plotted as filled symbols (filled circles=840 mm^3^ tumour volume and filled triangles=1280 mm^3^). For three of the four curves in Figure 4 (those in which tumour volumes are closely matched), a peak in EF5/Cy3 intensities is observed at the 140–160 μm zone. However, for the larger volume tumours (filled triangles), this peak is shifted to the left. During tumour growth, tumour cells tend to either outgrow or compress their own vasculature, leading to an overall increase in hypoxia (as evidenced by the upward shift in EF5/Cy3 intensities compared to the smaller volume controls) and necrosis. In support of such an increase in necrosis, the larger volume curve takes a downward turn at shorter distances from perfused blood vessels than does the smaller volume curve (since necrotic areas do not metabolize EF5, such regions will remain black and decrease median EF5/Cy3 intensities).

The effect of three daily doses of the anti-angiogenic agent, endostatin (20 mg/kg/day), is shown by the open circles. Here, a slight but insignificant improvement in both intravascular and tumour oxygenation is apparent. EF5/Cy3 intensities are similar for the zone immediately surrounding the blood vessels (1–20 μm distances), and these intensities increase with increasing distance from a vessel at approximately the same rate as for controls. The effect of a single dose of 30 Gy irradiation on this curve is quite different (as shown by the open triangles). Compared to volume-matched controls (filled circles), irradiated tumours had slightly lower intravascular oxygen levels (as evidenced by the intensities at the shortest distance zone), and the slope of the curve is substantially less steep. This decrease in slope in the irradiated tumours could be indicative of a decrease in oxygen consumption in the radiation-sterilized tumour cells or an increased proportion of quiescent cells.

## DISCUSSION

Both Eppendorf micro-electrode measurements and hypoxia marker uptake have been extensively used for gauging inter- and intratumoral variations in oxygenation. A disadvantage of the Eppendorf is that pO_2_ levels can only be obtained at discrete locations along the needle track (roughly every 400 μm), although multiple tracks can also be measured. In contrast, immunohistochemical staining of hypoxia markers permits microregional variations in hypoxia to be spatially mapped in two dimensions across an entire tumour section. By comparing the hypoxia marker images with corresponding images of vessel staining, proliferation markers, or angiogenic/anti-angiogenic cytokines, relationships among a variety of pathophysiological factors can also be correlated (Fenton *et al*, 2001b; Wijffels *et al*, 2001; Rijken *et al*, 2000; Zeman *et al*, 1993).

Although image analysis techniques are an indispensable aid in quantifying these types of images, automated counting procedures are not necessarily straight-forward. Perhaps the most difficult step in the analysis is the separation of countable objects from background using either image thresholding or colour segmentation. Previous studies have evaluated the effect of different threshold choices on hypoxic fraction, as determined by percentage hypoxia marker positive pixels (Rijken *et al*, 2000). These authors found that changing the threshold intensity by only two levels in either direction (in an image with 256 grey levels) resulted in a change of from 14–21% in the calculated hypoxic fraction for NITP staining. A change of four levels in the threshold intensity resulted in up to a 57% increase in the calculated hypoxic fraction. As these authors recommend, the selection of a constant threshold is essential when attempting to classify hypoxic tumours cells as simply positive or negative. An advantage of the current methods is that the hypoxia marker intensity levels of each pixel are also quantified. This means that the selection of an arbitrary threshold of hypoxia is not required. Constant threshold settings can be used for defining vascular structures, providing the fluorescent intensities or colours of the objects are sufficiently different from background (Vukovic *et al*, 2001; Rijken *et al*, 2000). However, images of immunostained sections can vary substantially under fluorescent and especially transmission microscopy, even within a given experiment. In such cases, manual thresholding or colour segmentation is often necessary for each image.

When analysing the intensities of fluorescently conjugated hypoxia markers, an essential first step is the calibration of the illumination source, as has been previously described in detail (Jenkins *et al*, 2000). To next quantify the distribution of hypoxia marker uptake, the most common approach has been to measure the percentage of the tumour section that is positively stained (Raleigh *et al*, 2001). Although this analysis is relatively straight-forward and corresponds to the notion of tumour hypoxic fraction, a substantial amount of information is not taken into account. Another approach has been to measure mean hypoxia marker intensities (Evans *et al*, 2001; Fenton, 2001; Fenton *et al*, 2001b), which again provides only an overall appraisal of changes in tumour hypoxia. Here, results can be especially misleading in the presence of large regions of necrosis, which do not metabolize EF5. Since hypoxia marker uptake has been clearly shown to depend proportionately on tissue pO_2_ levels (Jenkins *et al*, 2000; Koch *et al*, 1995), both intensity changes of the markers and their relationships to surrounding blood vessels are vital to fully describe microregional pathophysiological gradients.

Another potential problem in the automated analysis of vascular images lies in the determination of vascular density. The field shown in Figure 1B clearly includes five perfused blood vessels (based on the total vessel staining shown in Figure 1A). However, an automated count of this image would result in a much higher number of objects, corresponding to each of the discrete segments of each vessel. Although various image filtering operations can be utilized to close the gaps between these segments, such operations can also dramatically alter parameters such as vessel diameters and areas. Single vessels can also meander in and out of a thin frozen section and therefore be counted multiple times. Both of these difficulties are minimized by an alternative method for analysing vascular spacing, based on the perfused vessel distance map shown in Figure 1C. Following distance map filtering, individual pixel intensities are converted to levels directly proportional to the distances between tumour cells and the nearest blood vessel. If this distance map is next combined with a predefined image of white grid points (e.g., 50 μm spacing) on a black background using the image multiplication operator, the distance map intensities can be spatially sampled, which are directly proportional to the distribution of distances to the nearest vessel. This provides a histogram of the distribution of distances that oxygen and nutrients must diffuse to reach all points in the tumour, and an increase in this median distance thus corresponds to a decrease in vascularity. Although somewhat less intuitive than vascular density measures, the median of these distances provides a much more reliable method for quantifying changes in vascular spacing than does vascular density when using automated image analysis techniques.

It is possible that out-of-plane vessels may be an additional contributory factor to the decrease in hypoxia observed at higher distances from blood vessels. Since all images are based on two-dimensional slices through the tumours, vessels outside of the image plane are ignored. Thus EF5/Cy3 intensities could be locally reduced despite the absence of visible perfused vessels. Although this could potentially alter the relationship between EF5/Cy3 intensity and distance to the nearest vessel, such occurrences are expected, for the most part, to follow the distribution of visible vessels. Regions with high vascular densities will thus be more likely to have accompanying out of plane branches than regions of low vascular density. Although such out of plane branches would result in a decrease in absolute EF5/Cy3 intensities in associated regions, the effects should be similar for different treatment groups and are not expected to mask relative differences among groups.

The currently described zonal analysis of EF5/Cy3 intensities provides a comprehensive description of changes in tumour hypoxia as a function of distance to the nearest blood vessel. Compared with previous methods that utilize either dilation filters (Rijken *et al*, 2000) or square masks (Vukovic *et al*, 2001), these improved techniques permit the limits of each zone to be precisely defined based on a combination of distance mapping and thresholding operations. Although the presence of necrotic regions can have substantial effects on relative EF5/Cy3 intensities at increasing distances from vessels, these artifacts can be minimized if the analysis is limited to regions less than approximately 100–120 μm from blood vessels. This means that a laborious manual definition of necrotic regions from the H&E images is unnecessary if the zonal analysis is limited to this distance range. From this type of microregional analysis, relative changes in tumour cell oxygen consumption rates and intravascular oxygenation (which relates to vascular functionality) can both be estimated. Finally, by comparing changes in the distributions of hypoxic marker intensities around total *vs* perfused vessels, a relative index of the proportion of functional blood vessels in a given tumour can also be derived.
